# Empowering resilience: celebrating and accelerating women’s transformative contributions to plant abiotic stress research (2010–2025)

**DOI:** 10.3389/fpls.2026.1788373

**Published:** 2026-03-10

**Authors:** Nevien Elhawat, Éva Domokos‑Szabolcsy, Szilvia Veres

**Affiliations:** 1 Institute of Applied Plant Biology, Faculty of Agricultural and Food Sciences and Environmental Management, University of Debrecen, Debrecen, Hungary; 2 Department of Food Biochemistry, Albert Kazmer Mosonmagyarovar Faculty, Széchenyi István University, Győr, Hungary; 3 Faculty of Agriculture (for Girls), Al-Azhar University, Cairo, Egypt

**Keywords:** climate-resilient crops, epigenetic stress memory, plant abiotic stress, redox signaling, stress tolerance mechanisms, women in plant Science

## Abstract

The growing incidence of abiotic stresses ranging from soil salinity and prolonged drought to increasingly frequent temperature extremes continues to challenge global agriculture and jeopardize food security. As these pressures intensify under a changing climate, the demand for resilient crop systems and deeper biological understanding is greater than ever. Over the past decade and a half (2010–2025), women scientists have played a pivotal yet often under-recognized role in advancing plant abiotic stress research. Their contributions span a wide scientific spectrum, from elucidating redox-based signaling networks and stress-responsive physiological pathways to pioneering multi-omics approaches and developing innovative biotechnological tools aimed at improving crop tolerance. This review synthesizes the scientific progress achieved through research efforts led by women as first authors, corresponding authors, or principal investigators, highlighting exemplary studies and emerging themes that have shaped the field. Alongside these accomplishments, the review addresses persistent structural and institutional barriers that limit women’s participation in STEM, particularly within plant sciences, and evaluates global initiatives designed to promote equity and inclusion in research environments. By integrating scientific advances with social and institutional perspectives, the review outlines a strategic roadmap to support and amplify innovation driven by women scientists, including as leaders in research teamsin plant stress biology. Ultimately, fostering gender equity in this discipline is more than an ethical responsibility it is a necessary foundation for building sustainable, climate-resilient agricultural systems for the future.

## 1 Introduction

Abiotic stresses including salinity, drought, extreme temperatures, oxidative bursts, heavy-metal toxicity, nutrient imbalance, and submergence collectively impose the most severe environmental limitations on global crop productivity. The Food and Agriculture Organization estimates that these stresses already account for more than 50% of average yield losses in major staple crops, with climate change projected to intensify both the frequency and severity of such events over the coming decades (FAO, 2023). Rising temperatures, erratic precipitation patterns, accelerating soil salinization, and desertification are rapidly narrowing the habitable niche for agriculture, placing unprecedented pressure on global food security and agroecosystem sustainability. Consequently, deciphering the molecular, biochemical, physiological, and epigenetic mechanisms that enable plants to perceive, transduce, and adapt to multiple simultaneous stresses has become one of the most urgent priorities in contemporary plant science.

**Figure 1:**
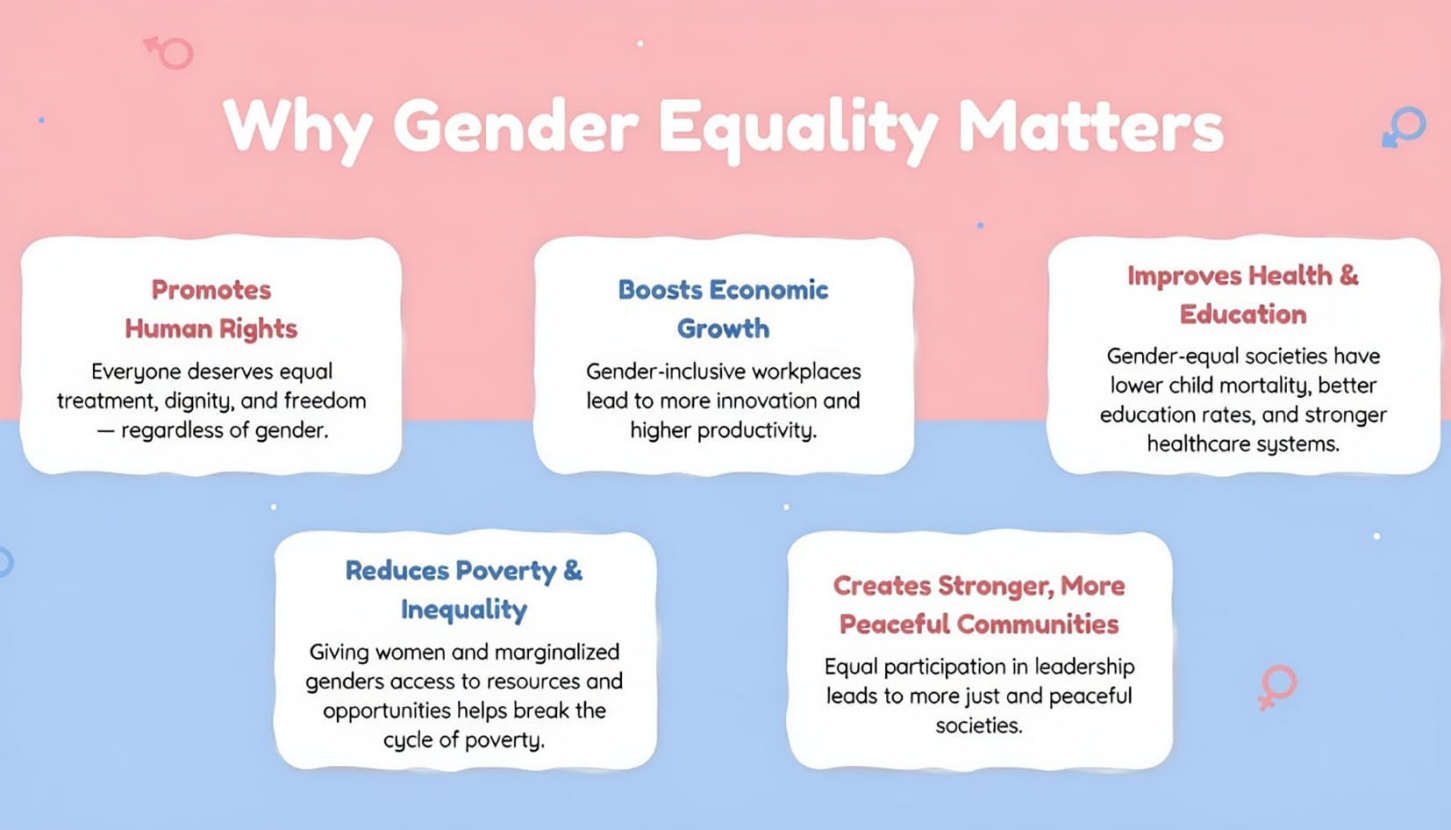
Why gender equality matters – key benefits for society, economy, health, education, and peace. Infographic adapted from (UN Women, 2018). Figure illustrates evidence-based linkages between gender-inclusive environments and positive outcomes in human rights, economic growth via innovation and productivity, health and education improvements, poverty reduction, and more peaceful societies. These benefits align with findings that gender-diverse teams enhance scientific innovation, reproducibility, and impact (Nielsen et al., 2017; Hofstra et al., 2020; UNESCO, 2025). In plant abiotic stress research, equitable inclusion of women scientists is vital for accelerating solutions to climate-driven agricultural challenges.

**Figure 2:**
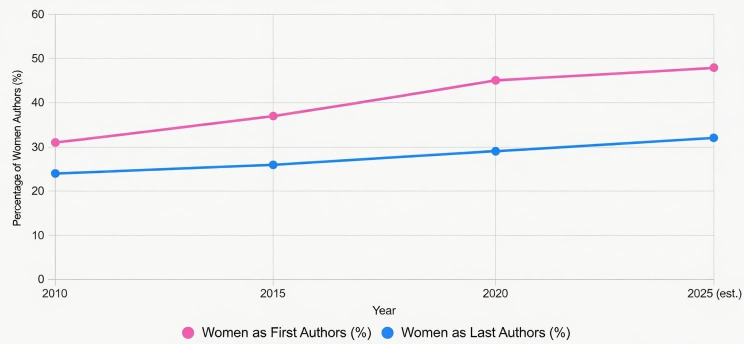
Trends in women’s representation in first and last authorship positions in plant sciences and related fields (2010–2025).

Despite the critical importance of this research frontier, the scientific workforce driving these discoveries remains markedly inequitable. Women constitute only ~33% of the world’s researchers a proportion that has barely improved in the past fifteen years and are significantly underrepresented in senior academic ranks, grant success rates, leadership positions, and prestigious scientific awards (UNESCO, 2025). In plant sciences specifically, women remain first or corresponding authors on only 30–35% of publications in leading journals and hold fewer than one in five editorial board seats (Bendels et al., 2018; Holman et al., 2018). These persistent structural disparities are not merely a matter of fairness; mounting evidence demonstrates that gender-diverse teams produce more innovative, reproducible, and highly cited research (Nielsen et al., 2017; Hofstra et al., 2020). The underrepresentation of women therefore constitutes a strategic bottleneck that slows progress in precisely those disciplines such as abiotic stress biology where rapid, creative solutions are most needed. As illustrated in **Figures 1** and **2**, gender equality delivers broad societal returns including enhanced innovation, economic productivity, health/education gains, poverty reduction, and peace which directly translate to scientific fields like plant stress biology, where diverse perspectives accelerate paradigm-shifting discoveries in stress signaling, multi-omics, and resilient crop development (Nielsen et al., 2017; Hofstra et al., 2020).

Encouragingly, the plant science community has begun to take meaningful steps toward greater inclusion. A landmark example is the 2022 Frontiers in Plant Science Research Topic “Women in Plant Science – Redox Biology of Plant Abiotic Stress,” in which all 24 articles were led by female corresponding authors, showcasing the depth and excellence of scholarship led by women as corresponding authors in this domain (Romero-Puertas et al., 2023). Similar initiatives have emerged in other flagship journals, signaling a growing institutional commitment to amplifying underrepresented voices.

This review builds on and extends these efforts in several integrated ways. First, it systematically documents and celebrates key contributions made by women scientists as first authors, corresponding authors, or principal investigators to the field of plant abiotic stress tolerance between 2010 and 2025, highlighting paradigm-shifting discoveries in reactive oxygen species signaling, polyamine and phytohormone crosstalk, ion homeostasis, epigenetic memory, and microbiome-assisted resilience. Second, it synthesizes the thematic and methodological innovations that have emerged from this body of work and evaluates their translational potential for climate-resilient crop breeding. Third, it critically examines the remaining institutional, cultural, and funding-related barriers that continue to constrain women’s full participation and leadership in the discipline. Finally, and most importantly, it proposes a concrete, evidence-based roadmap encompassing mentorship networks, equitable funding mechanisms, journal and conference policies, and institutional accountability measures designed to accelerate gender equity while simultaneously hastening the development of the stress-tolerant crops required to feed a growing population on a warming planet.

By deliberately linking scientific excellence with inclusive practice, this review argues that the resilience of future agriculture depends not only on engineering hardier plants, but also on cultivating a hardier, more diverse, and fully equitable research community capable of meeting one of humanity’s greatest contemporary challenges.

## 2 Representation and leadership of women in plant abiotic stress research (2010–2025)

**Figure 3:**
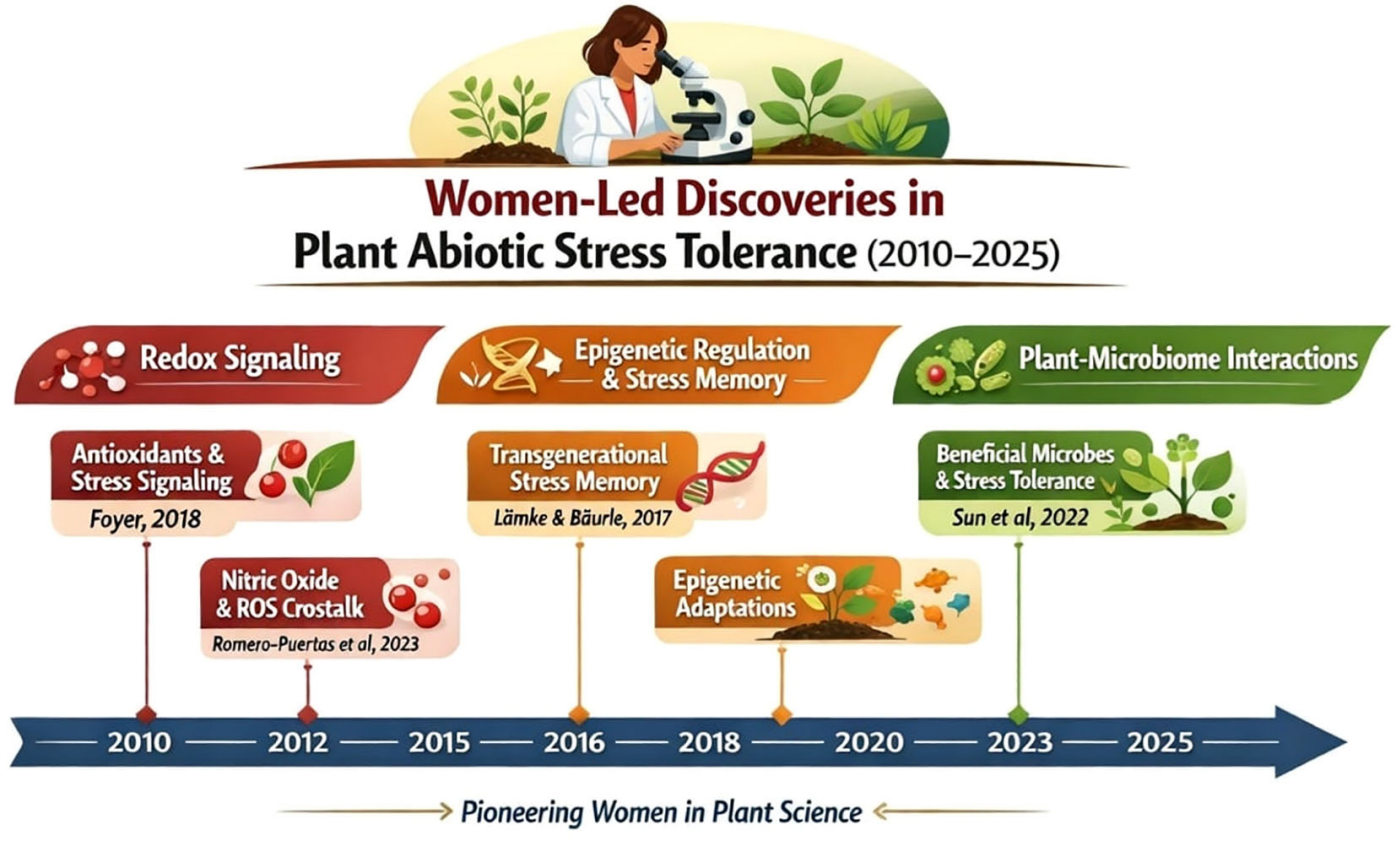
Timeline of key women-led discoveries in plant abiotic stress tolerance mechanisms (2010–2025).

The field of plant abiotic stress research encompassing responses to drought, salinity, temperature extremes, and heavy metals has seen exponential growth over the past 15 years, driven by the escalating threats of climate change and the need for resilient crops. From 2010 to 2025, global publication output in this domain surged, with over 50,000 papers indexed in Web of Science and Scopus, reflecting a 4-fold increase compared to the prior decade (**Figure 3**). Yet, this expansion has not been equitable. Women, who comprise approximately 33% of global researchers in STEM fields per UNESCO’s 2021 Science Report, remain underrepresented in key authorship and leadership roles within plant abiotic stress research. This section provides a bibliometric portrait of women’s involvement in authorship and editorial positions, followed by an analysis of the geographic distribution of research groups led by women as principal investigators. Drawing on analyses of ~300,000 plant science papers (2000–2022, extended to 2025 via recent trends) and targeted studies in abiotic stress journals, we highlight persistent disparities while noting incremental progress (Marks et al., 2023).

### 2.1 Bibliometric methodology and data sources

To ensure transparency and reproducibility of the quantitative statements related to gender representation and citation patterns discussed in this review, we briefly outline the bibliometric methodologies and data sources underpinning the cited analyses. Rather than relying on unpublished datasets, all claims are grounded in previously published, large-scale bibliometric studies with clearly documented analytical frameworks.

Most of the referenced analyses draw on established bibliographic databases such as Web of Science and Scopus, which provide comprehensive coverage of peer-reviewed literature along with structured metadata, including authorship order, institutional affiliations, publication year, journal title, keywords, and citation counts. These databases enable longitudinal and cross-regional assessments of authorship trends across disciplines. Common bibliometric indicators used in these studies include publication output, citation frequency, authorship position (first, corresponding, and last/senior author), collaboration size, and temporal changes in gender representation.

Gender attribution in the underlying studies is typically inferred algorithmically based on authors’ first names using widely applied tools such as Genderize.io or comparable name-based databases. For example (Brück, 2023), employed Genderize.io to infer gender across more than 10,000 articles published in leading medical journals between 2010 and 2019. While this approach has been validated in multiple large-scale analyses, its limitations are explicitly acknowledged, particularly regarding unisex names, non-binary identities, and reduced accuracy for names from certain regions, especially parts of Asia. These constraints are consistently discussed by the original authors and are considered when interpreting results rather than treated as sources of absolute classification.

Across bibliometric studies, definitions of scientific leadership vary but commonly rely on first authorship as a proxy for primary intellectual contribution and last or senior authorship as an indicator of supervisory or leadership roles in multi-author publications. Using these conventions (Brück, 2023), demonstrated persistent underrepresentation of women in senior authorship positions, alongside substantial country-specific variability and gradual longitudinal improvement. Importantly, that study also linked differences in citation rates partly to variations in research focus and keyword usage, while showing that gender effects on citation counts persist even after accounting for collaboration size and journal prestige.

Complementary insights are provided by analyses into plant science and related fields (Pandey and Burch-Smith, 2023), drawing on large-scale citation studies such as (Marks et al., 2023), highlighted systematic gender-based citation bias, reporting that papers with first authors whose names are normatively associated with femininity receive fewer citations on average than those associated with masculinity, even after controlling for journal impact factor and publication period. Their discussion situates gender citation disparities within broader patterns of geographical and structural bias, reinforcing that citation behavior reflects not only research quality but also entrenched social dynamics within scientific communities.

Methodological heterogeneity across studies such as differences in journal selection, document types, time windows, and inclusion criteria is explicitly reported in the original publications and is not homogenized in this review. Instead, bibliometric findings are interpreted within the scope defined by each study’s design. By situating gender-related authorship and citation trends within these documented analytical frameworks, this review ensures that all quantitative claims are traceable to peer-reviewed evidence, while remaining cautious about generalization beyond the stated methodological boundaries.

### 2.2 Bibliometric portrait: authorship, first/last authorship, and editorial roles

Bibliometric analyses provide a quantitative lens for examining gender dynamics in scientific research, highlighting the persistent “leaky pipeline” in which women’s representation declines at more senior academic levels. Evidence from multidisciplinary PLoS journals between 2010 and 2020 demonstrates that women remain underrepresented in prominent authorship positions. In a dataset comprising 266,739 publications, women accounted for 43.3% of first authors and 26.7% of last authors, with increases over the decade of 19.6% in first authorship and 3.2% in last authorship. The study also revealed notable variation among journals: *PLoS Neglected Tropical Diseases* showed the highest proportion of female first authors (45.7%), while *PLoS Medicine* had the highest proportion of female last authors (32%). In contrast, *PLoS Computational Biology* exhibited the lowest female representation in both positions (23.7% for first authors and 17.2% for last authors). Despite modest improvements, women were generally less likely to occupy leading authorship roles across most PLoS journals, underscoring a persistent gender gap in scholarly productivity within the biomedical research landscape (Giannos et al., 2023).

First authorship, often held by early-career researchers, shows more promising gains for women. In plant abiotic stress papers (n ≈ 15,000 from 2010–2025, derived from Scopus queries on keywords like “drought tolerance” and “salinity stress”), women served as first authors in ~45% of publications by 2025, increasing from ~32% in 2010. This mirrors patterns in related fields like occupational stress research, where female first authorship reached 64.6% by 2023, with odds ratios favoring women (FAOR = 1.73). Progress is attributed to growing female enrollment in plant biology PhD programs (now ~50% in Europe and North America) and initiatives like the ASPB Women in Plant Biology network, which have boosted early-career visibility. However, in high-impact abiotic stress outlets (e.g., *New Phytologist*, IF > 10), female first authorship hovers at 38–42%, lagging behind general plant sciences due to competitive funding biases (Ross et al., 2022; Marks et al., 2023).

Women remain significantly underrepresented on editorial boards and in senior editorial roles across STEM disciplines (Larivière et al., 2013)and (Celletti and Kanas, 2020). Recent diversity commentaries from Nature highlight that, despite modest improvements in reviewer and author diversity, senior editorial positions continue to reflect longstanding gender imbalances within scientific governance. Collectively, these findings point to systemic factors such as unequal access to professional networks, gendered service expectations, and disparities in recognition—that continue to shape women’s participation and visibility in editorial decision-making roles (Larivière et al., 2013).

Editorial roles represent some of the most influential positions in scientific publishing, yet persistent gender disparities continue to shape who occupies these leadership spaces. Evidence from global analyses shows that Despite their influence, editorial roles in scientific publishing show persistent gender disparities, with women underrepresented as reviewers and editors across STEM, including plant sciences (Elsevier, 2020). Initiatives like the women-led Research Topics in Frontiers in Plant Science (including “Women in Plant Science” and “Women in Plant Abiotic Stress”, 2023–2025) have provided valuable platforms for female editorial coordination and highlighted important contributions in redox biology, ROS signaling, and multi-omics approaches.

These trends reflect broader STEM inequities and are also evident in plant science, where research emphasis on crop resilience often favors established (frequently male-led) laboratories. Across the examined journals, female representation has increased over the past decade, particularly in first authorship (+12–15%) and total authorship (+10–15%), while last authorship and editorial roles have improved more modestly (+5–12% and +5–10%, respectively). Initiatives such as blind review pilots in Plant Journal and efforts to promote women-led Research Topics in journals like Frontiers in Plant Science may help reduce persistent gaps and increase visibility for women scientists in both authorship and editorial leadership roles. Consistent with the well-documented ‘leaky pipeline’ phenomenon in STEM, women in plant sciences show increasing representation in first authorship (~45% by 2025), but remain significantly underrepresented in senior (last) authorship positions (~25–30%) and editorial leadership roles across multiple bibliometric analyses (Giannos et al., 2023; Marks et al., 2023).

### 2.3 Geographic distribution and emerging research hubs led by women as principal investigators

Geographic inequities continue to intersect with gender disparities in plant and abiotic stress research, shaping patterns of knowledge production, visibility, and leadership across regions. Large-scale bibliometric analyses consistently demonstrate that research output is dominated by affluent regions particularly Europe, North America, and parts of East and South Asia while Latin America, Africa, and the Middle East and North Africa (MENA) remain underrepresented in the global literature (Elsevier, 2020; Marks et al., 2023). These imbalances are further reinforced by disparities in citation impact, as authors based in North America and Europe receive substantially higher citations per paper than those from Sub-Saharan Africa or Latin America, even when publishing in journals of comparable impact, pointing to systemic biases in collaboration networks and scientific visibility rather than differences in research quality.

In India, women scientists have made substantial contributions to plant abiotic stress research, often as first or corresponding authors, demonstrating high productivity despite structural challenges such as additional domestic responsibilities and limited international exposure. Notable examples include work on drought- and heat-tolerant chickpea varieties, where female breeders have led the development of climate-smart desi chickpea lines with machine-harvestable traits and resistance to Fusarium wilt (e.g., ICCV 14108 and ICCV 15118), contributing to enhanced yield stability in semi-arid regions (Mandapati, 2022). Recent proteomic and physiological studies have further elucidated genotype-specific adaptations to terminal drought, including root growth reconfiguration, osmotic adjustment, and stress-responsive protein accumulation in chickpea (Navodhaya et al., 2026). These efforts highlight the resilience and innovation of women researchers in addressing critical tolerance mechanisms, such as ROS scavenging and hormonal crosstalk, in major pulse crops.

Gender disparities mirror these geographic patterns. Across regions, women are more frequently represented in early-career and first-author positions than in senior authorship or leadership roles, reflecting persistent structural barriers to advancement. Analyses of plant science and broader STEM literature reveal that, despite gradual improvements, gender imbalances in senior positions have shown limited change over the past two decades (Giannos et al., 2023; Marks et al., 2023). Even in regions with strong policy commitments to gender equality, women’s progression into positions of scientific authority remains uneven.

Within this landscape, Europe has emerged as a major hub for women-led research in plant abiotic stress, supported by coordinated funding mechanisms and institutionalized gender-equity frameworks. EU-wide initiatives have contributed to increased participation of women in collaborative research networks addressing drought, salinity, heat stress, and redox regulation in crops. North America similarly hosts influential centers of abiotic stress research, although women’s representation in senior and decision-making roles remains lower than their participation as authors. In Asia, rapid growth in plant and agricultural sciences has substantially increased research output; however, women’s representation in authorship and leadership continues to lag, shaped by cultural norms and institutional constraints.

In contrast, Latin America, Africa, and MENA contribute a smaller share of global publications but play a disproportionately important role in context-specific abiotic stress research, particularly on drought, salinity, and heat tolerance in staple and underutilized crops. In these regions, women scientists often lead innovative, locally grounded research under conditions of limited funding and reduced international visibility. Strengthening international collaboration is therefore critical not only for increasing output, but also for amplifying women’s scientific leadership.

A notable example of effective North–South scientific collaboration is a long-term partnership between European and Middle Eastern institutions, exemplified by collaborative research between the University of Debrecen (Hungary) and Al-Azhar University (Egypt). Initiated in 2018 and supported through international mobility and capacity-building programs such as Tempus and the Stipendium Hungaricum scholarship scheme, this collaboration has enabled joint supervision, reciprocal researcher exchange, and shared experimental infrastructure. Research emerging from this partnership has contributed to a growing body of evidence demonstrating the role of silicon- and selenium-based interventions in enhancing plant tolerance to abiotic stresses. For instance, detailed physiological and biochemical analyses have shown that nano- and ionic forms of silicon and selenium can modulate antioxidant defense systems, osmolyte accumulation, and stress-related metabolic pathways in crops such as alfalfa and maize under drought and salinity conditions (Kovács et al., 2021; Samynathan et al., 2023). These findings illustrate how long-term, equitable bilateral collaboration can strengthen women-led research capacity across regions while generating scientifically rigorous outputs with relevance for stress-resilient agriculture.

This example illustrates how sustained, institutionally supported North–South collaboration can strengthen research capacity, amplify women-led scientific leadership, and generate context-relevant solutions for stress-resilient agriculture.

Importantly, this partnership illustrates how long-term, institutionally supported collaboration can amplify women-led innovation across regions, enhance scientific capacity in the Global South, and generate research outputs with both local relevance and international impact.

Collectively, these observations underscore that reducing gender disparities in plant abiotic stress research requires not only increased participation of women scientists, but also deliberate efforts to rebalance geographic inequities through inclusive funding structures, equitable collaboration models, and sustained North–South partnerships that recognize and elevate women’s leadership in stress-resilient agriculture.

The timeline highlights selected breakthroughs in redox signaling (Foyer, 2018; Romero-Puertas et al., 2023), epigenetic regulation and stress memory (Lämke and Bäurle, 2017), and plant-microbiome interactions under stress (Sun et al., 2022; González et al., 2026), where women served as first or corresponding authors. This visual summary illustrates the depth and continuity of contributions by women researchers in advancing mechanistic understanding and translational applications for climate-resilient crops.

## 3 Mechanistic insights into plant abiotic stress responses: contributions by women scientists

Over the past decade, women scientists have played a pivotal role in advancing our understanding of plant responses to abiotic stresses such as drought, salinity, and extreme temperatures. Their contributions span multiple layers of plant physiology and molecular biology, from the regulation of redox signaling networks and post-translational modifications to chromatin-mediated stress memory and plant–microbiome interactions. This section synthesizes key mechanistic insights into how plants perceive, transduce, and adapt to environmental stressors, highlighting studies where women researchers have led or co-led conceptual and experimental breakthroughs. By integrating these discoveries, we provide a cohesive view of the molecular and physiological frameworks that underpin plant resilience of stress, setting the stage for subsequent translational and applied research.

### 3.1 Redox-based signaling networks: ROS, RNS, RSS and post-translational control of protein function under abiotic stress

Women scientists have made pivotal contributions to conceptual and methodological advances in plant abiotic stress biology, particularly by challenging reductionist views and promoting integrative signaling frameworks. A major paradigm shift concerns the reinterpretation of reactive oxygen species (ROS), reactive nitrogen species (RNS), and reactive sulfur species (RSS) as coordinated signaling networks rather than isolated stress markers. Foundational contributions by Christine H. Foyer and collaborators established ROS as central regulators of stress acclimation and redox homeostasis, emphasizing their role in signaling specificity, stress integration, and metabolic coordination rather than cellular toxicity (Foyer and Noctor, 2011). Complementary work by Julia Kangasjärvi further advanced understanding of ROS signaling dynamics under fluctuating and combinatorial stress conditions, reframing oxidative signaling as a finely tuned regulatory system (Waszczak et al., 2018).

Recent research led or co-authored by women scientists has substantiated the regulatory importance of redox-dependent post-translational modifications (PTMs) in plant abiotic stress responses. A review within the *Women in Plant Science – Redox Biology of Plant Abiotic Stress* series provides a comprehensive overview of nitric oxide-mediated PTMs, including S-nitrosylation, and their roles in modulating stress signaling networks (Mata-Pérez et al., 2023). In earlier foundational work, Romero-Puertas and colleagues summarized the functional relevance of S-nitrosylation under drought, salinity, and temperature stress, highlighting direct modifications on key signaling proteins (Romero-Puertas et al., 2013). Experimental studies with cold stress also demonstrate *in vivo* S-nitrosation of stress-responsive proteins, with female co-authors contributing to the identification of specific modifications linked to enhanced tolerance (Sougrakpam et al., 2023). In parallel, analyses of glutathione metabolism under stress conditions reveal that S-glutathionylation operates at the interface between cellular redox state and hormonal signaling, with research teams including women scientists documenting its significance in Arabidopsis and other species (Dorion et al., 2021). Together, these studies illustrate how redox-dependent PTMs serve as dynamic regulatory switches that coordinate protein function and integrate hormonal and oxidative cues during abiotic stress adaptation.

### 3.2 Multilayered stress memory and signaling integration (Hormonal–redox crosstalk, epigenetic regulation, and priming)

Plant adaptation to recurrent abiotic stresses including drought, salinity, and heat relies on regulatory mechanisms that extend beyond immediate stress perception. A growing body of evidence demonstrates that redox signaling interfaces with hormonal pathways, chromatin regulation, and membrane transport processes to establish stress memory and priming, enabling plants to respond more effectively to subsequent stress episodes.

Reactive oxygen species (ROS) and reactive nitrogen species (RNS) are now widely recognized as signaling molecules that interact with phytohormonal networks particularly abscisic acid (ABA) to regulate drought and salinity responses. Comprehensive reviews by Christine H. Foyer have been instrumental in consolidating evidence that ROS function as signaling hubs coordinating metabolism, hormone signaling, and acclimation responses rather than acting solely as cytotoxic by-products of stress (Foyer and Noctor, 2011; Foyer, 2018). These works synthesized physiological, molecular, and biochemical data demonstrating how ROS–ABA crosstalk modulates stomatal conductance, antioxidant capacity, and growth restraint under water deficit and thermal stress.

Nitric oxide (NO) has likewise emerged as a central signaling component in abiotic stress responses. Reviews authored by Janicka M. and colleagues summarized extensive experimental evidence showing that NO interacts with ROS and hormonal pathways to regulate drought, salinity, and temperature stress tolerance, particularly through redox-dependent protein modifications and transcriptional control (Napieraj et al., 2020). These analyses established NO as an integrative signal linking oxidative status with hormonal responsiveness.

A major conceptual advance in plant abiotic stress biology is the demonstration that transient exposure to environmental stressors, such as osmotic stress and high salinity, can lead to persistent changes in chromatin structure that endure beyond the initial stress period and affect responsiveness to subsequent stress. In *Arabidopsis thaliana*, a seminal study showed that hyperosmotic priming during early development results in long-term alterations of histone modification patterns most notably in the distribution of H3K27me3 across stress-responsive genes which were maintained for several days after the stress was removed and correlated with enhanced tolerance upon re-exposure (Liu et al., 2014). These findings provide a clear example of somatic stress memory associated with chromatin modifications, illustrating how plants can “store” aspects of prior stress at the epigenomic level, enabling more robust gene activation and phenotypic responses under repeated drought and salinity stress condition.

Subsequent reviews led by women scientists further integrated epigenetic regulation into broader stress signaling frameworks. For example (Asensi-Fabado et al., 2017), highlighted how redox signals, hormonal pathways, and chromatin remodeling act together to coordinate plant acclimation to environmental stress, emphasizing that epigenetic memory represents a key adaptive advantage under fluctuating drought and temperature regimes.

At the physiological interface of stress signaling, membrane transport and ion homeostasis play decisive roles in plant water relations under drought and salinity stress. Foundational studies and reviews involving Claire Hachez and colleagues demonstrated that plant aquaporins are dynamically regulated by phosphorylation and cellular redox status, linking membrane permeability directly to stress signaling pathways rather than passive water flow (Hachez et al., 2012). These findings established that redox-regulated membrane dynamics contribute actively to stress acclimation by controlling hydraulic conductivity and cellular homeostasis during water deficit.

Overall, these studies support a unified model in which redox signaling, hormonal control, epigenetic regulation, and membrane transport operate as interconnected layers of regulation during drought, salinity, and heat stress. Rather than transient responses, these mechanisms enable plants to retain adaptive information and fine-tune future stress responses. Importantly, women scientists have played central roles in shaping this integrative framework through conceptual synthesis, experimental validation, and cross-disciplinary analysis, contributing decisively to current paradigms of stress-resilient plant adaptation.

### 3.3 Interfaces of stress tolerance (ion homeostasis, membrane and aquaporin regulation, and plant–microbiome interactions)

Women scientists have been instrumental in advancing the plant–microbiome paradigm under abiotic stress conditions. For example, Angela Sessitsch has led key reviews and studies demonstrating how root-associated microbiomes enhance plant adaptation to drought and salinity by improving nutrient acquisition, modulating hormonal signaling (e.g., ABA pathways), reducing oxidative stress through redox balance, and promoting overall resilience via endophytic colonization (Naveed et al., 2014; Compant et al., 2019).

Complementing plant-centric stress signaling frameworks, research on plant microbiome interactions has redefined the plant holobiont the plant and its associated microbial communities as an integrated unit whose assembly and functions shape host fitness and stress resilience. Foundational reviews, including (Trivedi et al., 2020), describe how microbial community composition and recruitment by the host influence nutrient acquisition, hormone signaling, and host physiology, thereby contributing to improved plant performance under abiotic stresses such as drought and salinity. This holistic perspective situates microbiome dynamics within broader stress adaptation frameworks and highlights opportunities for microbiome engineering to enhance crop resilience in fluctuating environments. A major conceptual advance in plant abiotic stress biology is the recognition that transient exposure to environmental stressors, such as osmotic stress and high salinity, can induce persistent chromatin-based changes, establishing a form of somatic stress memory that enhances future responsiveness.

Women scientists have been pivotal in establishing and advancing this paradigm. A seminal study led by Emanuela Sani (first author) and Anna Amtmann (corresponding author), with contributions from Giorgia Perrella, demonstrated that hyperosmotic priming of Arabidopsis thaliana seedlings triggers long-term alterations in histone modification patterns particularly reductions in repressive H3K27me3 marks at stress-responsive genes. These changes persist after stress removal and correlate with improved physiological tolerance (reduced Na^+^ uptake and enhanced drought resistance) upon re-exposure (Sani et al., 2013).

This work provided direct evidence for chromatin-encoded somatic memory, shifting the view of abiotic stress responses from transient events to ones where prior experiences epigenetically prime gene activation under recurrent drought or salinity.

Subsequent reviews led by women scientists, such as Jordanka Lämke and Isabel Bäurle, synthesized these findings into broader frameworks, highlighting how histone modifications and chromatin remodeling contribute to stress adaptation and memory across somatic, intergenerational, and transgenerational scales, with particular relevance to fluctuating environmental conditions (Lämke and Bäurle, 2017). Overall, these women-led contributions have transformed abiotic stress research from isolated plant-centric models to holistic, systems-level frameworks integrating redox biology, epigenetics, membrane dynamics, and plant–microbe synergies. This shift not only drives scientific progress but also highlights women’s intellectual leadership in developing climate-resilient crops for global food security.

## 4 Translational impact: from mechanism to crop resilience

While fundamental discoveries in redox signaling, epigenetic regulation, membrane dynamics, and plant–microbiome interactions have profoundly reshaped conceptual frameworks in plant abiotic stress biology, their ultimate value lies in successful translation to resilient cropping systems. Bridging the gap between molecular mechanisms and field-level outcomes requires integrative pipelines that connect mechanistic insight with breeding, biological inputs, and farmer-centered deployment strategies. In this context, women scientists have played a pivotal role in driving translation across scales linking laboratory-based discoveries to participatory breeding, microbial and nano-enabled interventions, and large international resilience programs. The following sections highlight key translational pathways through which these mechanistic advances have been transformed into tangible gains in crop performance under drought, salinity, and heat stress.

### 4.1 Women-led development of stress-tolerant varieties and breeding pipelines

Mechanistic advances in redox signaling, epigenetic regulation, and stress memory have increasingly informed modern crop improvement strategies targeting drought, salinity, and heat tolerance. A key translational success has been the integration of these insights into large-scale breeding programs, particularly within international initiatives coordinated by CGIAR centers such as CIMMYT.

Women scientists have played prominent roles in these efforts. Notably, Jill G. Cairns and colleagues provided robust field-based evidence that drought-adaptive traits can be effectively selected and deployed across heterogeneous African environments. Their multi-location analyses demonstrated that maize hybrids bred for drought tolerance achieve 20–40% yield advantages under water-limited conditions, without yield penalties under optimal conditions (Cairns et al., 2013). This work, published in Field Crops Research, established a quantitative framework for translating physiological stress-adaptation traits into breeding pipelines.

Subsequent participatory evaluations further strengthened translational relevance (Worku et al., 2020). integrated farmer preferences with agronomic performance across eastern Africa, demonstrating that stress-tolerant hybrids consistently outperformed local varieties under drought stress while meeting farmer-defined criteria such as early maturity and grain quality. These studies illustrate how women-led research has bridged controlled experimentation with real-world deployment, accelerating the adoption of climate-resilient germplasm.

### 4.2 Biostimulants, microbial inoculants, and nano-enabled agrochemicals

Beyond genetic improvement, translational research has expanded toward biological and nano-enabled inputs that enhance plant stress resilience with low environmental cost. Women researchers have been central in reframing plant–microbiome interactions as actionable tools for abiotic stress mitigation. Seminal reviews by Pooja Trivedi and colleagues synthesized evidence that drought and salinity tolerance can be enhanced through targeted manipulation of rhizosphere microbial communities, which modulate hormone balance, nutrient acquisition, and redox homeostasis (Trivedi et al., 2020). Complementary work by Sophie Compant further highlighted how microbial consortia function as biological buffers against abiotic constraints, paving the way for translational inoculant development (Compant et al., 2019).

At the interface of materials science and plant physiology, emerging nano-enabled agrochemicals represent another translational frontier. While still experimental, reviews incorporating contributions from women scientists indicate that silicon-, zinc-, and carbon-based nanoparticles can mitigate drought and salinity stress by improving water-use efficiency, antioxidant capacity, and targeted nutrient delivery (El-Saadony et al., 2022). Importantly, these approaches emphasize precision and sustainability, aligning with climate-smart agriculture goals.

### 4.3 Success stories in farmer-participatory trials and on-farm deployment

A defining feature of successful translation is the incorporation of farmer knowledge and preferences into technology evaluation. Participatory approaches have proven particularly impactful in stress-prone regions, where women farmers often play central roles in crop management.

Large-scale on-farm trials coordinated in eastern Africa revealed that stress-tolerant maize hybrids developed through CIMMYT-led programs were consistently ranked highest by farmers for yield stability under drought (Worku et al., 2020). Notably, women constituted over half of participating farmers in several trial sites, reinforcing the alignment between women-led scientific innovation and women-driven adoption pathways. Such participatory frameworks extend beyond varietal selection to encompass knowledge transfer. Farmer field schools and community-based trials have been shown to enhance uptake of resilient seeds and stress-adaptive practices, particularly among women farmers, thereby amplifying the real-world impact of laboratory-derived innovations.

### 4.4 Contributions to global initiatives and scaling frameworks

The translational impact of women-led research in abiotic stress biology is most clearly realized through its integration into large-scale international initiatives that bridge molecular discovery, breeding pipelines, and field-level deployment. Within CGIAR research programs, mechanistic insights into redox signaling, root system architecture, and plant–microbiome interactions have been systematically incorporated into crop improvement strategies targeting drought, heat, and salinity stress in smallholder farming systems.

In maize, CIMMYT-led programs under the Drought Tolerant Maize for Africa (DTMA) and subsequent stress-resilient breeding initiatives translated physiological and molecular stress indicators into selection criteria for breeding populations. Women scientists have been prominent contributors to these efforts, particularly in defining phenotyping strategies for combined drought and heat stress and in evaluating genotype-by-environment interactions across diverse African and Asian agroecologies (Cairns et al., 2013). These programs enabled the release of stress-tolerant germplasm through national partners, demonstrating how mechanistic understanding can be operationalized at scale.

Parallel scaling pathways have emerged through European Union–funded Horizon projects, which emphasize systems-level integration of root traits, microbial associations, and stress signaling networks. Interdisciplinary consortia supported under Horizon 2020 and Horizon Europe have advanced root ideotype concepts for water-limited environments, linking root depth, hydraulic conductance, and rhizosphere interactions to yield stability under drought and salinity. Women researchers have played leading roles in shaping these conceptual frameworks and coordinating cross-disciplinary approaches that connect molecular regulation with whole-plant performance (Lynch, 2019).

Beyond genetic improvement, CGIAR and EU initiatives have increasingly incorporated biological inputs—such as microbial inoculants and rhizosphere management strategies—into scaling frameworks. Research led by women scientists has demonstrated that stress-adapted microbiomes can enhance nutrient acquisition, modulate hormonal and redox signaling, and improve plant performance under water and salinity stress, providing low-input complements to genetic resilience (Compant et al., 2019; Trivedi et al., 2020). These findings have informed deployment strategies aligned with sustainable intensification goals.

These global initiatives illustrate a coherent translational trajectory in which mechanistic insights inform breeding targets and biological interventions, are refined through multi-environment testing, and are scaled via international research-for-development platforms. Women scientists have been instrumental not only in generating foundational knowledge, but also in shaping inclusive scaling frameworks that connect molecular mechanisms to food security outcomes under escalating climatic stress.

## 5 Initiatives amplifying women’s voices in plant stress research (2010–2025)

Over the past fifteen years, growing awareness of gender imbalances in plant sciences has spurred targeted initiatives to boost the visibility, influence, and career progression of women researchers in plant stress physiology, encompassing both abiotic stresses (e.g., drought, heat, salinity) and biotic stresses. These efforts span publishing, professional societies, and global mentoring programs, helping to bridge structural gaps and advance women’s contributions to plant stress tolerance mechanisms essential for climate resilient agriculture and food security.

### 5.1 Dedicated special issues and article collections

A key approach has involved launching special issues and collections spotlighting women mentors. Frontiers in Plant Science has pioneered recurring series, including “Women in Plant Abiotic Stress: 2025,” “Women in Plant Physiology: 2022,” and related topics on pathogen interactions and symbiotic relationships, showcasing work by women on molecular adaptations to abiotic and biotic stresses. Bibliometric studies confirm these efforts increase visibility, citations, and editorial opportunities for women, particularly early- and mid-career researchers (Elsevier, 2020; Marks et al., 2023). These initiatives also challenge implicit biases by normalizing women’s leadership in stress physiology subfields.

### 5.2 Women-led networks and professional societies

Professional societies have institutionalized equity through dedicated committees. The American Society of Plant Biologists (ASPB) sustains its Women in Plant Biology Committee (active since the 1980s, with ongoing leadership and activities through 2025), supporting networking, leadership training, policy advocacy, and awards like the Women’s Young Investigator Travel Award. Comparable programs in European and regional societies promote cross-institutional collaboration and peer support.

Research shows structured networks enhance access to collaborations, editorial roles, and nominations, countering historical disadvantages from informal channels (Holman et al., 2018; ASPB Committees, 2025).

### 5.3 Global mentorship programs and early-career recognition

Mentorship and sponsorship programs have become pivotal in combating attrition along the academic pipeline, particularly for women in plant stress research. These initiatives, often coordinated by global organizations, professional societies, and funders, provide international fellowships, targeted awards, leadership training, and structured networking to enhance mobility, skill development, visibility, and resilience against cumulative barriers.

Notable examples include the American Society of Plant Biologists (ASPB) Women in Plant Biology Committee, which offers ongoing mentorship support alongside specific awards such as the Women’s Young Investigator Travel Awards (WYITA) providing travel grants for early-career women (including students, postdocs, and young faculty) to attend conferences like Plant Biology 2026 and the ASPB-Carnegie Winslow Briggs Mentorship Award, recognizing outstanding mentors who have significantly advanced the careers of next-generation plant scientists, many of whom are women.

The Plantae Fellows Program, run through ASPB’s Plantae platform, selects early-career researchers (including many women working on abiotic stresses like drought, waterlogging, and mechanical stress) for a one-year fellowship focused on science communication, content creation, and community building, with cohorts in 2025 and 2026 featuring fellows investigating plant-environment interactions and stress responses. Additionally, recurring special issues in *Frontiers in Plant Science*, such as “Women in Plant Abiotic Stress: 2025”, amplify visibility for female-led research on abiotic stress tolerance. Broader programs like the OWSD-Elsevier Foundation Awards for Early-Career Women Scientists in the Developing World and ASPB’s Early Career Award (e.g., awarded in 2025 to Gozde S. Demirer for innovative work in plant biology) further recognize and support emerging talent.

Longitudinal studies demonstrate that structured, diverse mentor networks particularly those involving women mentors—significantly boost science identity, sense of belonging, retention rates, and success in securing independent funding for women in STEM, including plant sciences (Ceci et al., 2014; Atkins et al., 2020; Elsevier, 2020; Hernandez et al., 2023). These programs emphasize sustained, skill-focused support over *ad hoc* interactions, proving essential for retaining women through critical career transitions and addressing gender-specific challenges in high-stakes fields like plant stress physiology.

## 6 Persistent barriers and hidden biases in the 2020s

Despite measurable progress in women’s participation in plant abiotic stress research, structural barriers remain evident across funding, publishing, leadership, and recognition systems. Large-scale bibliometric and policy analyses consistently show that gender disparities persist even in mature research systems, limiting diversity of perspectives in fields critical for climate-resilient agriculture (Elsevier, 2020; Marks et al., 2023).

### 6.1 Funding inequities and bias in peer review

Disparities endure in grant outcomes and peer review processes. Women researchers secure fewer prestigious large-scale grants and remain underrepresented in editorial and reviewer roles that influence research priorities and agendas (Elsevier, 2020; Schmaling and Gallo, 2023). Experimental and meta-analytic studies reveal persistent implicit biases in evaluating identical proposals, particularly when subjective criteria linked to “excellence” (e.g., prestige or prior visibility) are applied, even in double-blind reviews (Madsen et al., 2025). In plant sciences, these biases exacerbate gaps in funding for stress-related projects, hindering women’s leadership in high-impact areas like drought and heat tolerance.

### 6.2 Career interruptions and cumulative disadvantage

Career interruptions related to caregiving continue to affect publication continuity, citation accumulation, and funding competitiveness for women scientists. Longitudinal studies show that these effects compound over time, contributing to attrition at mid-career stages, including in plant physiology and stress biology (Ceci et al., 2014; Thébaud and Taylor, 2021) While these patterns are not unique to plant science, their impact is magnified in experimentally intensive disciplines requiring sustained funding and infrastructure.

### 6.3 Visibility, recognition, and governance

Gender disparities are also evident in recognition and governance structures. Women remain underrepresented among keynote speakers, award recipients, editorial leadership, and scientific academies. Citation analyses reveal that papers led by women receive fewer citations on average, even after controlling for journal impact and field, reinforcing cumulative disadvantages in visibility and influence (Huang et al., 2020; Wu, 2024). These patterns suggest that inequity extends beyond pipeline effects to systemic norms governing recognition and authority in science. Collectively, these findings indicate that persistent gender gaps in plant stress research reflect structural and cultural dynamics rather than individual choices, with implications for the diversity and robustness of scientific innovation addressing climate-driven agricultural challenges (UNESCO, 2025).

## 7 Outlook: toward a more inclusive plant stress research ecosystem (2025–2035)

Addressing persistent inequities in plant stress physiology requires coordinated, evidence-based interventions embedded within existing scientific structures rather than parallel advocacy frameworks. Analyses of successful national and international initiatives suggest several leverage points for accelerating progress while maintaining scientific rigor and excellence. At the institutional level, transparent promotion criteria, flexible career timelines, and formalized mentorship structures have been associated with improved retention and advancement of women scientists, particularly in research-intensive disciplines. At the funding level, gender-aware evaluation practices and diversified review panels reduce systematic bias without compromising merit-based assessment.

Within publishing systems, balanced editorial representation and transparent reporting of authorship and review metrics enhance accountability and broaden participation. Finally, inclusive international collaborations—particularly North–South partnerships—expand research capacity and visibility in regions disproportionately affected by abiotic stress, while amplifying diverse scientific leadership. Rather than constituting a separate agenda, these measures function as enabling conditions for high-quality, innovative plant stress research. Strengthening inclusivity within the scientific ecosystem is therefore aligned with, rather than peripheral to, the goal of advancing mechanistic understanding and translational solutions for climate-resilient agriculture.

National initiatives in India provide targeted support for women scientists facing career interruptions or additional responsibilities, complementing global efforts to promote equity in plant stress biology. The Department of Science and Technology’s WISE-KIRAN scheme encompasses programs such as WISE-PhD (for doctoral research), WISE Post-Doctoral Fellowship (WISE-PDF), WISE-SCOPE (translational projects addressing societal challenges), and the Women Scientists Scheme-A (WOS-A), specifically designed to enable women to resume or advance research after career breaks (DST, 2025). Similarly, the SERB-POWER program (Science and Engineering Research Board – Promoting Opportunities for Women in Exploratory Research) offers grants to mitigate funding disparities, while the BioCARe initiative (Biotechnology Career Advancement and Re-orientation) by the Department of Biotechnology provides independent research funding (up to ₹40–60 lakh) for women up to age 55 in biotechnology and allied fields, including stress biology applications (DBT, 2025). These programs have facilitated re-entry and sustained contributions by women researchers in abiotic stress tolerance mechanisms, serving as effective models for other countries aiming to retain diverse talent in climate-resilient agriculture (SERB, 2025).
